# A Mendelian Randomization Analysis to Expose the Causal Effect of IL-18 on Osteoporosis Based on Genome-Wide Association Study Data

**DOI:** 10.3389/fbioe.2020.00201

**Published:** 2020-03-20

**Authors:** Ni Kou, Wenyang Zhou, Yuzhu He, Xiaoxia Ying, Songling Chai, Tao Fei, Wenqi Fu, Jiaqian Huang, Huiying Liu

**Affiliations:** ^1^Department of Oral Prosthodontics, School of Stomatology, Dalian Medical University, Dalian, China; ^2^School of Life Science and Technology, Harbin Institute of Technology, Harbin, China

**Keywords:** genome-wide association studies, Interleukin-18, Osteoporosis, casual effect, Mendelian randomization

## Abstract

Accumulating evidence showed that Interleukin (IL) level is associated with Osteoporosis. Whereas, most of these associations are based on observational studies. Thus, their causality was still unclear. Mendelian randomization (MR) is a widely used statistical framework that uses genetic instrumental variables (IVs) to explore the causality of intermediate phenotype with disease. To classify their causality, we conducted a MR analysis to investigate the effect of IL-18 level on the risk of Osteoporosis. First, based on summarized genome-wide association study (GWAS) data, 8 independent IL-18 SNPs reaching genome-wide significance were deemed as IVs. Next, Simple median method was used to calculate the pooled odds ratio (OR) of these 8 SNPs for the assessment of IL-8 on the risk of Osteoporosis. Then, MR-Egger regression was utilized to detect potential bias due to the horizontal pleiotropy of these IVs. As a result of simple median method, we get the SE (−0.001; 95% CI−0.002 to 0; *P* = 0.042), which means low IL-18 level could increases the risk of the development of Osteoporosis. The low intercept (0; 95% CI −0.001 to 0; *P* = 0.59) shows there is no bias due to the horizontal pleiotropy of the IVs.

## Introduction

Osteoporosis is a chronic disease with a variety of causals to bone mineral density and bone loss of quality (Sambrook, 2006). Since the bone weakening in Osteoporosis patients, it increases the risk of a broken bone and other diseases among the elderly (Tu et al., 2018). Bone is a type of living tissue, which is constantly being broken down and replaced. When the creation of new bone doesn't keep up with the loss of old one, Osteoporosis occurs (Seo et al., 2018; Papaleontiou et al., 2019; Wang et al., 2019). Osteoporosis affects all the countries including men and women. For example, about 90,000,000 Osteoporosis patients in china, which covers the 7.1% of the total population. Since lots of complications affected by Osteoporosis and its incurability, it is very important for preventing the Osteoporosis. Whereas, there are no typical symptoms in the early stages of bone loss according to the current knowledge (Hennemann, 2002). This raises the difficulties for prevention. To solve this problem, it is urgent to expose the causal clinical phenotypes of Osteoporosis.

Interleukin (IL) is a class of cytokines, which is produced by a variety of cells and also functions on a variety of cells (Kato and Perl, 2018; Zhang et al., 2018). Current, about forty types of IL was discoveries in human body. IL-18 is one of them, which locates at 11q22.2-22.3. IL-18 is powerful inflammatory cytokines, the most characteristic feature of which is the regulation of cellular proliferation and differentiation (Weiss et al., 2018; Youssef et al., 2018; Prencipe et al., 2019; Valero et al., 2019). Recent studies show that IL-18 plays important roles in immune regulation, resistance to infection and anti-tumor. Furthermore, IL-18 has been identified as the causal of multiple chronic diseases, such as type 2 diabetes (Zou et al., 2018).

The relationship between IL and Osteoporosis has been investigated in observational studies decades of years. Early in 1993, Lewis et al. investigated a transgenic mice with disorder in bone homeostasis that inappropriately express the cytokine IL-4 (Lewis et al., 1993). And then they observed that Osteoporosis was associated with the IL-4. In 2005, Rusinska et al. evaluated the relationship between multiple ILs and the etiopathogenesis of idiopathic osteoporosis in children (Rusinska and Chlebnasokół, 2005). In 2010, Edwards et al. exposed the relationship between IL-6 and rheumatoid arthritis-associated osteoporosis (Edwards and Williams, 2010).

**Figure 1:**
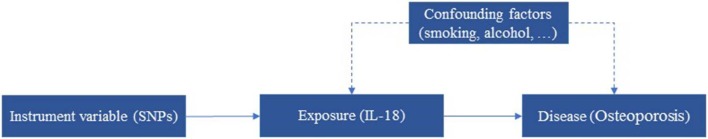
MR analysis using SNPs as instrumental variables for estimating the influence of IL-18 on the risk of Osteoporosis.

Although current advantages on investigating the relationship between IL-18 and Osteoporosis, it is still not clear whether IL-18 is the consequent or causal effect of the Osteoporosis. This is the common problem for many associations between phenotypes and diseases. With the development of Genome-Wide Association Studies (GWAS) and identification of molecular mechanism in recent years (Li et al., 2015; Zhou et al., 2017a,b; Tang et al., 2018; Tan et al., 2019), Mendelian randomization (MR) analysis is widely used to expose the causal effect of phenotypes on the development of diseases. For example, body mass index and C-reaction protein are identified as the causal effect of the development of type 2 diabetes (Cheng et al., 2018b, 2019c). Meanwhile, some negative associations are also exposed, such as associations between infant length and type 2 diabetes (Zhuang et al., 2019a). As other statistical analysis and machine learning methods (Du et al., 2018; Liao et al., 2018; Wang B. et al., 2018; Wang L. et al., 2018; Cheng et al., 2019b; Han et al., 2019; Lv et al., 2019; Yang et al., 2019; Zeng et al., 2019; Zou and Ma, 2019; Zhao et al., 2020), MR is an instrumental variable (IV) based method for inferring associations between phenotypes and diseases. As shown in Figure 1, genetic variants as SNPs are often used as IVs. This is because that SNPs are genetic characterize and occurred before phenotypes and diseases, which can avoid reverse causality. Here Z (e.g., SNPs) represents IVs, X is phenotype (e.g., IL-18), and Y is the disease (Osteoporosis). To conduct MR analysis, the IVs should meet two assumptions. One is that SNPs should be robustly associated with phenotype (IL-18), and the other is that SNPs can influence the disease only through the phenotype.

To explore the causal effect of IL-18 on the development of Osteoporosis, we conducted a MR analysis in this study. First, we defined a framework for processing data to establish IVs for MR analysis. Next, simple median method was used for calculating the pooled result based on IVs. Then, to avoid bias and analysis the limitation of our method, MR Egger analysis and leave-one-out validation was conducted.

## Materials and Methods

Summary-level data of GWAS dataset was the fundamental for MR analysis. IVs should be extracted from IL-8 related GWAS dataset. And the further analysis need Osteoporosis related GWAS dataset. To meet the MR assumptions and reduce the bias, the summarized GWAS data was processed. Subsequently, MR analysis involving simple median method, leave-one-out validation, MR-Egger analysis was conducted to comprehensively assess the causal effect of IL-8 on the risk of the development of Osteoporosis.

### Summarized GWAS Data for IL-18

In 2014, Matteini et al. (2014) performed a genome-wide association study (GWAS) on Cardiovascular Health Study and InCHIANTI cohorts. Totally, it contains 1200 InCHIANTI cohorts and 3200 CHS population. After genotyping, they used GWAPower software to assess the difference in power of the combined InCHIANTI-CHS meta-analysis compared to single study analyses. Then, significant SNPs of IL-18 were identified and used for analyzing causal relationship of IL-18 on the risk of Type 2 Diabetes (Zhuang et al., 2019a). Here, we extracted SNPs, effect allele (EA), allele frequencies, beta coefficients, and standard errors (SEs) as summarized data.

### Summarized GWAS Data for Osteoporosis

In 2018, Bycroft et al. published their prospective cohort study (Bycroft et al., 2018), which contains approximately 500,000 individuals from across the United Kingdom, aged between 40 and 69 at recruitment. It also provided detailed description and summarized data of deep genetic and phenotypic data. We downloaded summarized GWAS data of Osteoporosis from UK Biobank, which involves susceptibility loci together with other SNPs, beta coefficient, EA, SEs and their *P*-values, and etc. Totally, it contains 933 cases and 360,261 controls.

### Data Processing

We process the summarized GWAS data for constructing IVs of MR analysis. Here IVs are genetic variants. Each SNP should be significantly associated with IL-18, and should be not associated with Osteoporosis. Thus, we extracted SNPs significant associated with IL-18, and then removed these SNPs associated with Osteoporosis. We defined *P* < 5^*^10^−8^ as significant associated SNPs of IL-18, and we defined *P*-value more than 0.05 as not associated SNPs of Osteoporosis. To avoid over-precise estimates due to genetic pleiotropy, we should remove these SNPs with potential linkage disequilibrium (LD) relationships. The analogous method has been used in the MR analysis of causal effect of phenotype on T2DM (Cheng et al., 2019c; Zhuang et al., 2019b). To remove SNPs with LDs, we ranked significant SNPs of IL-18 based on *P*-values. For each SNP, we removed those SNPs in LD with it (*R*^2^ threshold of 0.001) or within 10,000 kb physical distance based on a reference dataset (Devuyst, 2015). This process was iterated for each of significant SNPs of IL-18.

### MR Analysis

MR analysis contains simple median method, leave-one-out validation, MR-Egger regression analysis (Bowden et al., 2015). Simple median method was used for assessing the pooled influence of IL-18 on the risk of Osteoporosis. Leave-one-out validation was performed for assessing sensitivity of each of IVs. Egger regression analysis was used to evaluate pleiotropy bias of IVs.

- Simple median method

Simple median method was described in the previous study (Burgess et al., 2016) for evaluating the influence of clinical phenotype on the risk of disease, which is defined as following equation.

(1) $$\begin{array}{l} beta_{XY}=beta_{ZY}/beta_{ZX}, \end{array}$$

where *X, Y*, and *Z* are IL-18, Osteoporosis, and IVs, respectively, Wald ratio (*beta*_*XY*_) of IL-18 to Osteoporosis through specified IV, *beta*_*ZY*_ is the per-allele *log(OR)* of Osteoporosis from summarized GWAS data of Osteoporosis. *beta*_*ZX*_ is the per-allele *log(OR)* of IL-18 from summarized GWAS data of IL-18. Then, we calculated SE of association between IL-18 and Osteoporosis for each Wald ratio, which is defined as Equation 2.

(2) $$\begin{array}{l} SE_{XY}=SE_{ZY}/SE_{ZX}, \end{array}$$

where *SE*_*ZY*_ and *SE*_*ZX*_ represent the *SE* of the IV-Osteoporosis and IV-IL-18 associations from corresponding summarized GWAS data, respectively. Next, we calculated 95% confidence intervals (CIs) from the *SE* of each Wald ratio. To get the pooled influence of these IVs, simple median method was used as meta-analysis for estimating comprehensive influence of IVs.

- Leave-one-out validation

We used leave-one-out validation for evaluating the sensitivity of each of IVs as following. To assess the influence of a SNP of IVs to the pooled result, we remove this SNP from IVs to get the result using simple median method. Thus, the corresponding result is obtained without considering this SNP. The fluctuation of the pooled results before and after removing the SNP could reflect the sensitivity of this SNP. This process was iterated on each of these IVs to get the influence for each of IVs.

- MR-Egger analysis

We conducted a MR-Egger regression analysis about asymmetry test to measure bias based on potential pleiotropic effect of IVs (Bowden et al., 2015). The MR-Egger regression is source from Egger regression, which is designed for detecting bias due to small study and pleiotropy in meta-analysis. Here, MR-Egger used intercept as an estimated value for evaluating the average pleiotropic effect of IVs. For example, the larger or smaller an intercept, the more of pleiotropy effect should be. All statistical tests for this study were undertaken using the R Package of MRBase (Hemani et al., 2018).

## Results

### Genetic Variants as IVs

Totally, 8 SNPs were extracted as significant associated SNPs of IL-18. Those SNPs were not associated with Osteoporosis and have no LD associations. As a result, those 8 significant SNPs of IL-18 were eventually selected as IVs for the MR analysis, which were shown in the Table 1. Each line of the table documents 10 items involving the SNP, EA, chromosome position, beta coefficients and SE of the SNP on the risk of IL-18 and Osteoporosis, and so on.

**Table 1:** Associations of genetic variants with IL-18 and Osteoporosis.

**SNP**	**EA (frequency)**	**Gene ID/Symbol**	**chr**	**Position**	**IL-18_beta**	**IL-18_SE**	**Beta**	**se**	***p-*value**
rs7577696	G (0.49381)	NA	2	32278782	0.08	0.01	8.71E-05	6.15E-05	1.57E-01
rs6760105	G (0.491214)	6683 (SPAST)	2	32307386	0.06	0.01	8.63E-05	6.15E-05	1.61E-01
rs6748621	C (0.495208)	84661 (DPY30)	2	32262201	0.08	0.01	1.01E-04	6.17E-05	1.03E-01
rs2300702	C (0.545527)	6716 (SRD5A2)	2	31788018	0.07	0.01	−9.89E-05	6.11E-05	1.06E-01
rs2268797	C (0.552716)	6716 (SRD5A2)	2	31783752	0.07	0.01	−9.53E-05	6.11E-05	1.18E-01
rs2250417	T (0.304113)	83875 (BCO2)	2	32412832	0.1	0.01	−3.32E-05	6.01E-05	5.81E-01
rs212745	C (0.480232)	55676 (SLC30A6)	2	32457537	0.07	0.01	−9.38E-05	6.16E-05	1.28E-01
rs212713	C (0.494409)	58484 (NLRC4)	11	112085316	0.06	0.01	−9.82E-05	6.00E-05	1.02E-01

### The Causal Effect of IL-18 on the Risk of Osteoporosis

**Figure 2:**
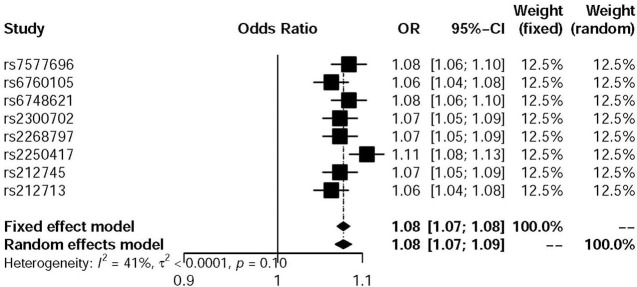
Forest plot of Wald ratios and 95% CIs of IVs.

After using 8 individual SNPs as IVs for MR analysis based on two summary-level GWAS data, we used simple median method for pooled analysis. Figure 2 shows that there is no evidence of heterogeneity between variants of the summarized data. As a result, we get the SE (−0.001; 95% CI −0.002 to 0; *P* = 0.042), which means low IL-18 level could increase the risk of the development of Osteoporosis.

### Sensitivity Analysis for Individual SNPs

**Figure 3:**
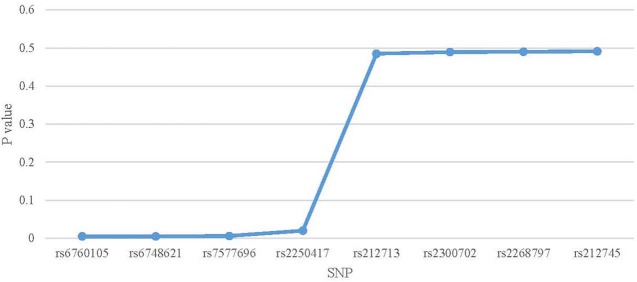
Scatter plot of the *P*-values in leave-one-out analysis.

Figure 3 shows estimate result of the leave-one-out analysis. After removing rs6760105, rs6748621, rs7577696, or rs2250417 from 8 IVs, the estimate value shows small fluctuation. And the result is consistent with using all the IVs. This means that this four SNPs activate weak influence to the estimate result. In comparison, after removing rs212713, rs2300702, rs2268797, or rs212745, estimate value shows large fluctuation. This means that this four SNPs activate strong influence to the estimate result. The detailed information about leave-one-out validation result is shown in Table 2.

**Table 2:** Results based on leave-one-out validation.

**SNP (leave out)**	**Wald ratio**	**95%CI lower**	**95%CI upper**	***p*-value**
rs6760105	−0.001	−0.002	0	0.005
rs6748621	−0.001	−0.002	0	0.005
rs7577696	−0.001	−0.002	0	0.006
rs2250417	−0.001	−0.002	0	0.02
rs212713	0	−0.001	0.001	0.485
rs2300702	0	−0.001	0.001	0.489
rs2268797	0	−0.001	0.001	0.49
rs212745	0	−0.001	0.001	0.491

### Pleiotropic Effect Analysis for IVs

**Figure 4:**
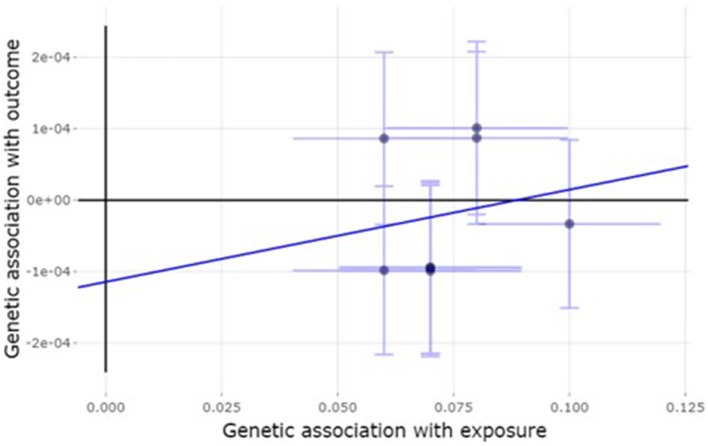
The estimate of horizontal pleiotropy based on MR-Egger analysis.

Figure 4 shows the effect estimate based on MR-Egger regression. The low intercept (0; 95% CI −0.001 to 0; *P* = 0.59) shows there is no bias due to the horizontal pleiotropy of the IVs.

## Discussion

Till now, it is not clear that the level of IL-18 is the causal or consequence of the development of Osteoporosis. To expose the relationship, we conducted an MR analysis based on two summarized GWAS datasets in this study. As a result of simple median method, we get the SE (−0.001; 95% CI −0.002 to 0; *P* = 0.042). This means that low IL-18 level could increase the risk of the development of Osteoporosis.

Observational study is a widely used way to reveal the associations between phenotypes and diseases. Whereas, it couldn't be used for exposing the causal effect. With the increase of GWAS data and abundance of molecular characterize (Cheng, 2019; Cheng et al., 2019a; Dao et al., 2020; Zhang et al., 2020), more and more researchers choose MR analysis for this purpose. MR analysis is an IV-based framework, which requires summarized GWAS data. In recent years, MR analysis has helped us to identify lots of causal effects, such as body mass index and C-reactive protein increase the risk of type 2 diabetes (Cheng et al., 2019c; Zhuang et al., 2019b). Here, the number of the case and control for GWAS data is very important for the estimation. In the previous study, the summarized data of IL-18 GWAS data has been applied in exposing the relationship between IL-18 and T2DM (Zhuang et al., 2019a). And the number of case and control is over 5,000. In addition, the UK biobank provided 933 Osteoporosis and 360,261 controls. The number of the samples for IL-18 and Osteoporosis GWAS data is the baseline for our MR analysis.

To make the MR analysis reliably, the summarized GWAS data was processed strictly to choose suitable IVs. First, significant associated SNPs of IL-18 (*P* < 5^*^10^−8^) was extracted. Then, these Osteoporosis (*P* < 5^*^10^−2^) associated SNPs were removed. This process is to make sure the IVs meet the requirement of MR's assumption. In addition, to reduce the bias of IVs due to the pleiotropic effect, SNPs in IVs with LDs were removed. In actually, the comprehensive effect of IVs are used for estimating the causal effect. The SNPs with LDs should be deemed as a SNP to reduce the bias based on the replication. To detect the potential bias due to the horizontal pleiotropy of IVs, MR egger method was conducted. As a result, we got the low intercept (0; 95% CI −0.001 to 0; *P* = 0.59), which shows there is no bias due to the horizontal pleiotropy of the IVs. All the data process of IVs is to reduce the bias, and make the result more reliably.

Although there is no direct associations between IVs and Osteoporosis, we also validated their potential linkages. We downloaded associated genes of Osteoporosis from a widely used functional annotation database OAHG (Cheng et al., 2016, 2018a), and compared them with genes that IVs located at (Table 1). Up to 440 genes of Osteoporosis were documented in OAHG, and no intersection between them and genes of IVs. This validate further that no potential bias of IVs based on current knowledge.

Although MR analysis show the advantages in distinguishing causal effect from general associations. It also has the limitations. With the incensement of samples, the summarized data of phenotype and disease should be a little alteration. This could lead to the changes in fluctuations about summarized data of GWAS data and even in IVs. This would influence the estimated result. Thus, to avoid this problem, the huge number of samples is very important. In this study, we do a leave-one-out analysis to judge which SNPs could influence our results. As a result, we find out SNPs rs6760105, rs6748621, rs7577696, or rs2250417 shows a little capability in influencing the result. In comparison, SNPs rs212713, rs2300702, rs2268797, or rs212745 could largely influence the result, all of which are located at genes SRD5A2, SLC30A6, and NLRC4. With the increase of the GWAS data, the impact of these SNPs could be decreased.

In addition to simple median method, IVW method is another frequently used method for MR analysis. As a result of IVW method, we get SE (0; 95% CI −0.001 to 0.001; *P* = 0.625), which is inconsistent with the result of simple median method. Currently, most of current MR analysis based on different methods often show inconsistent results. Each method has its advantages and limitations. In general, the causal effect could be validated when the relationship is supported by one of these methods. To provide more reliable validation, it needs randomized controlled trial (RCT). Whereas, it is very hard to conduct RCT. Thus, most of current causal effect between clinical phenotypes and diseases are validated based on MR analysis. Although current success in applying MR analysis, it couldn't substitute for RCT.

In summary, 8 SNPs were used as IVs for estimating the causal effect of IL-18 on the development of Osteoporosis. Results show that low IL-18 level could increase the risk of the development of Osteoporosis based on simple median method. In considering the limitation of MR method and current samples of GWAS data, further experiment for the conclusion is expected.

## Data Availability Statement

Publicly available datasets were analyzed in this study. This data can be found here: http://biobank.ctsu.ox.ac.uk/crystal/field.cgi?id=20002.

## Author Contributions

HL conceived and designed the experiments. NK and WZ analyzed the data. NK, WZ, and HL wrote the manuscript. All authors read and approved the final manuscript.

### Conflict of Interest

The authors declare that the research was conducted in the absence of any commercial or financial relationships that could be construed as a potential conflict of interest.
